# PAPP-A proteolytic activity enhances IGF bioactivity in ascites from women with ovarian carcinoma

**DOI:** 10.18632/oncotarget.5010

**Published:** 2015-08-24

**Authors:** Jacob Thomsen, Rikke Hjortebjerg, Ulrick Espelund, Gitte Ørtoft, Poul Vestergaard, Nils E. Magnusson, Cheryl A. Conover, Trine Tramm, Henrik Hager, Claus Høgdall, Estrid Høgdall, Claus Oxvig, Jan Frystyk

**Affiliations:** ^1^ Medical Research Laboratory, Department of Clinical Medicine, Faculty of Health, Aarhus University, DK-8000 Aarhus C, Denmark; ^2^ Department of Gynecology, Aarhus University Hospital, DK-8200 Aarhus N, Denmark; ^3^ Department of Endocrinology and Internal Medicine, Aarhus University Hospital, DK-8000 Aarhus C, Denmark; ^4^ Division of Endocrinology and Metabolism, Endocrine Research Unit, Mayo Clinic, Rochester, MN 55905, USA; ^5^ Department of Pathology, Aarhus University Hospital, DK-8000 Aarhus C, Denmark; ^6^ Clinic of Gynecology, Juliane Marie Centret, Rigshospitalet, DK-2100 Copenhagen, Denmark; ^7^ Department of Pathology, Herlev University Hospital, DK-2730 Herlev, Denmark; ^8^ Department of Molecular Biology and Genetics, Faculty of Science & Technology, Aarhus University, DK-8000 Aarhus C, Denmark

**Keywords:** IGF-I, PAPP-A, KIRA assay, IGFBP-4, malignant ascites

## Abstract

Pregnancy-associated plasma protein-A (PAPP-A) stimulates insulin-like growth factor (IGF) action through proteolysis of IGF-binding protein (IGFBP)-4. In experimental animals, PAPP-A accelerates ovarian tumor growth by this mechanism. To investigate the effect of PAPP-A in humans, we compared serum and ascites from 22 women with ovarian carcinoma. We found that ascites contained 46-fold higher PAPP-A levels as compared to serum (*P* < 0.001). The majority (80%) of PAPP-A was enzymatically active. This is supported by the finding that ascites contained more cleaved than intact IGFBP-4 (*P* < 0.03). Ascites was more potent than serum in activating the IGF-I receptor (IGF-IR) *in vitro* (+31%, *P* < 0.05); in 8 of 22 patients by more than two-fold. In contrast, ascites contained similar levels of immunoreactive IGF-I, and lower levels of IGF-II (*P* < 0.001). Immunohistochemistry demonstrated the presence of IGF-IR in all but one tumor, whereas all tumors expressed PAPP-A, IGFBP-4, IGF-I and IGF-II. Addition of recombinant PAPP-A to ascites increased the cleavage of IGFBP-4 and enhanced IGF-IR activation (*P* < 0.05). In conclusion, human ovarian tumors express PAPP-A, IGFBP-4 and IGFs and these proteins are also present in ascites. We suggest that both soluble PAPP-A in ascites and tissue-associated PAPP-A serve to increase IGF bioactivity and, thereby, to stimulate IGF-IR-mediated tumor growth.

## INTRODUCTION

Insulin-like growth factor I (IGF-I) and its primary target, the IGF-I receptor (IGF-IR) stimulate malignant transformation, tumor progression and metastasis [1–4]. This also holds true for ovarian carcinoma, where up-regulated IGF-I and IGF-IR expression has been demonstrated in surgical specimens from patients with advanced stages as well as with poorly differentiated ovarian tumors [5]. These clinical observations gain support from experimental studies. *In vitro*, IGF-IR activation was essential for transformation of normal ovarian epithelial tissue into cancer tissue and for maintenance of this pathological phenotype [6]. *In vivo*, studies in nude mice demonstrated that transfection of ovarian mesothelial cells with the human IGF-IR gene renders the cells tumorigenic and enable them to form large debilitating tumors as opposed to untransfected cells [6]. Conversely, silencing of IGF-IR expression with siRNA suppressed tumor growth in mice injected with the ovarian cancer cell line OVCAR3 [7]. Thus, clinical as well as pre-clinical data support a pathogenic role for IGF-I and the IGF-IR in the development and progression of ovarian carcinoma.

Recently, we studied the IGF system in non-malignant ascites from patients with alcoholic liver cirrhosis [8]. We observed that the ability of ascites to activate the IGF-IR *in vitro* (i.e. bioactive IGF) was fourfold higher than that of serum [8]. This finding may be of relevance for patients with ovarian carcinoma as production of ascites is a frequent complication [9]. Therefore, with the notion in mind that ascites *per se* is a negative prognostic factor [10, 11] and that the advancement of disease is related to IGF-IR activation [1–3, 6, 7, 12], we compared the ability of malignant ascites and serum from women with ovarian cancer to activate the IGF-IR *in vitro*. To this end, we collected serum, ascites and tumor tissues from 22 women with ovarian carcinoma, 19 of whom were newly diagnosed, and made a detailed analysis of the IGF system. Serum from age-matched healthy controls was included as well. Our analysis included pregnancy-associated plasma protein-A (PAPP-A), which stimulates IGF action through proteolysis of IGFBP-4 [13, 14]. In experimental models of ovarian carcinoma, PAPP-A has been demonstrated to enhance IGF activity and accelerate tumor growth [12–15], whereas PAPP-A neutralization has been shown to reduce tumor growth and delay the formation of ascites [16].

## RESULTS

### Circulating levels of IGF system components in ovarian cancer patients vs. healthy women

The IGF system is responsive to systemic disease [17]. Therefore, we compared the circulating IGF system in ovarian cancer patients to that in healthy woman (Table 1). The comparison showed that patients had borderline reductions in bioactive IGF (*P* = 0.09) and total IGF-II (*P* = 0.06), significant reductions in total IGF-I (*P* = 0.005), pro-IGF-II (*P* < 0.001) and IGFBP-3 (*P* < 0.001), and significant increases in PAPP-A (*P* = 0.03) and IGFBP-2, the latter being more than 7-fold elevated (*P* < 0.001).

**Table 1:** Levels of IGF related peptides in serum/EDTA plasma and ascites from 22 women suffering from ovarian cancer and 15 age-matched healthy women Data are median and quartiles. Circulating levels in patients and controls were compared by the Mann-Whitney rank sum test. In patients, circulating vs. ascites levels were compared by the Wilcoxon signed rank test.

IGF related peptide	Circulating levels in controls	Circulating levels in patients	Ascites levels in patients	Ratio between ascites and serum in patients	Circulating levels in controls vs. patients *P* value	Circulating vs. ascites levels in patients *P* value
Bioactive IGF (μg/l)	1.39 [1.04–1.58]	1.11 [0.82–1.33]	1.33 [0.87–2.68]	1.31 [0.83–2.55]	0.09	<0.05
Total IGF-I (μg/l)	78 [59–97]	57 [43–72]	57 [46–81]	1.11 [0.93–1.35]	0.005	0.13
Total IGF-II (μg/l)	491 [413–581]	437 [371–487]	211 [158–250]]	0.45 [0.41–0.63]	0.06	<0.001
Pro-IGF-II (μg/l)	149 [129–176]	109 [72–141]	52 [41–74]	0.52 [0.39–0.66]	<0.001	<0.001
IGFBP-2 (μg/l)	181 [130–191]	1339 [868–1920]	2030 [1373–2954]	1.44 [1.07–2.74]	<0.001	<0.001
IGFBP-3 (μg/l)	3697 [3348–4379]	2596 [2096–3323]	1560 [1325–2131]	0.67 [0.57–0.76]	<0.001	<0.001
PAPP-A (μg/l)	0.67 [0.57–0.83]	0.83 [0.74–1.10]	51.5 [30.0–57.9]	46 [36–79]	<0.05	<0.001
Intact IGFBP-4 (μg/l)*	n.d.	259 [177–288]	85 [32–108]	0.35 [0.21–0.50]	—	<0.001
C-terminal IGFBP-4 (μg/l)*	n.d.	n.d.	129 [70–220]	—	—	<0.03**
IGFBP-2 degradation (%)	0 [0–0]	5 [0–11]	9 [0–13]	—	<0.001	<0.05
IGFBP-3 degradation (%)	3 [0–5]	52 [29–89]	36 [6–89]	—	<0.001	NS

All measurements were performed in serum and ascites. The only exceptions were intact and C-terminal IGFBP-4, which require EDTA plasma instead of serum. Due to lack of EDTA plasma, C-terminal IGFBP-4 was not determined (n.d.) in patients. For the same reason, intact and C-terminal IGFBP-4 levels were not assessed in controls. NS: not significant;

* : *n* = 18

** : C-terminal IGFBP-4 vs. intact IGFBP-4 in ascites.

Immunoblotting (not shown) demonstrated that patient sera contained a modest fraction of degraded IGFBP-2 (*P* < 0.001) and a substantial fraction of degraded IGFBP-3 (*P* < 0.001), whereas the cleavage products of these IGFBPs were almost completely absent in controls.

### Comparison of levels of IGF system components in ascites and serum from patients with ovarian cancer

**Figure 1:**
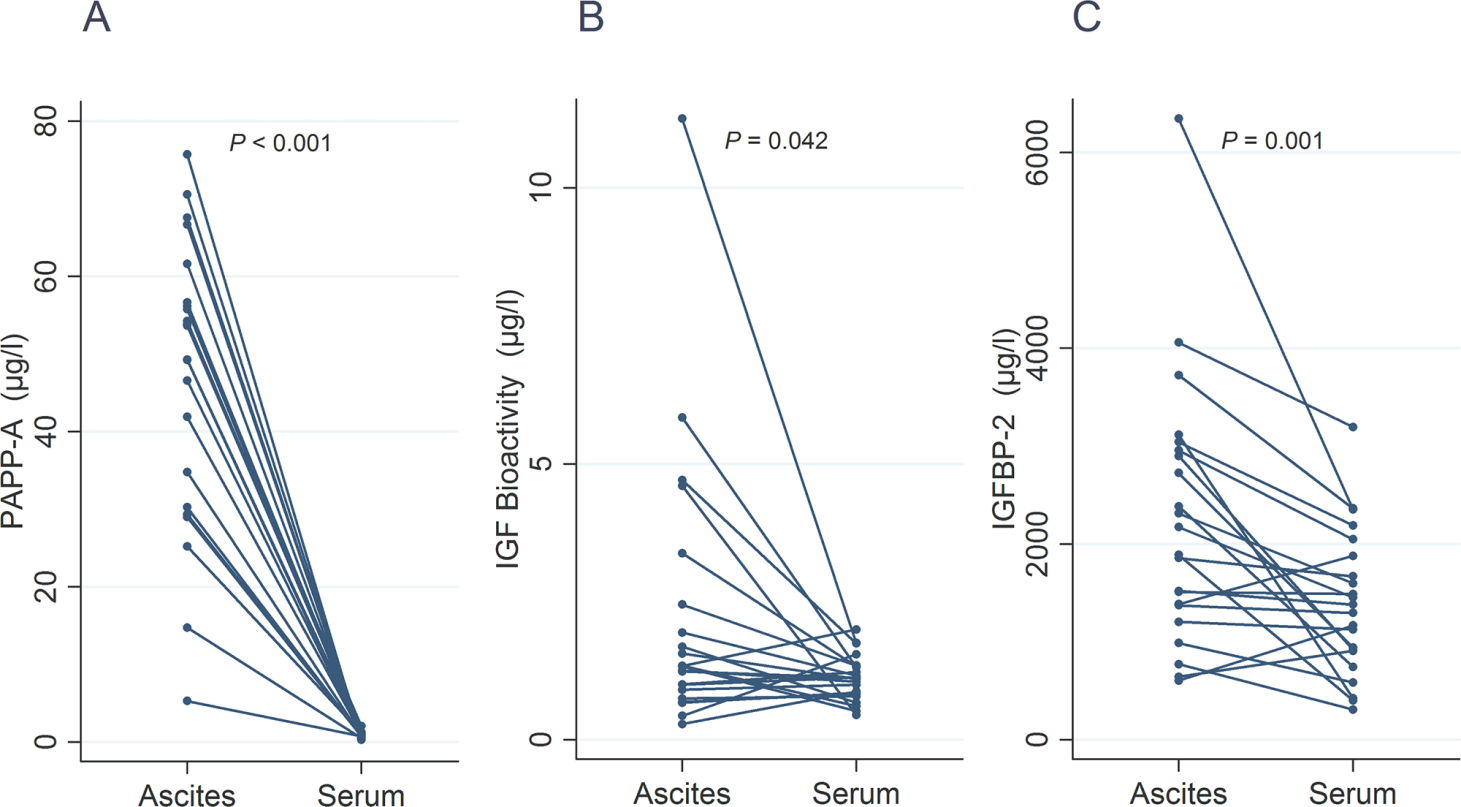
Line plots illustrating individual values in ascites and serum of PAPP-A (A), bioactive IGF (B), and IGFBP-2 (C)

The most pronounced difference between ascites and serum from the patients was observed for PAPP-A, which was 46-fold higher in ascites than serum (*P* < 0.001). Paired individual values of PAPP-A in serum and ascites are shown in Figure 1A.

The immunoassay for PAPP-A does not allow for distinction between enzymatically active PAPP-A, which corresponds to free, dimeric PAPP-A, and enzymatically inactive PAPP-A composed of dimeric PAPP-A covalently linked to two molecules of eosinophil major basic protein (proMBP) [18, 19]. Therefore, the enzymatic activity of PAPP-A in ascites was determined by measuring the total concentration of PAPP-A and the concentration of PAPP-A/proMBP complex [20]. PAPP-A activity was then expressed as the fraction of PAPP-A not complexed to proMBP. Our results suggested that the majority of PAPP-A present in ascites was enzymatically active, as the fraction of inactivated, proMBP-bound PAPP-A constituted only 20 [14–26] % of total PAPP-A.

In accordance with our finding of elevated levels of active PAPP-A in ascites, this compartment contained less intact IGFBP-4 than plasma (*P* < 0.001). Further, ascites contained a higher concentration of proteolytically cleaved IGFBP-4 than intact IGFBP-4 (*P* < 0.03). This was demonstrated by specific measurements of intact IGFBP-4 and the C-terminal cleavage product of IGFBP-4.

The ability of ascites to activate IGF-IR *in vitro* (i.e. a measure of bioactive IGF) was increased in ascites by 31% as compared to serum (*P* < 0.05). In eight patients, the ascites IGF bioactivity was more than twofold higher than the corresponding serum value. Paired individual values of bioactive IGF in serum and ascites are shown in Figure 1B. In contrast, levels of immunoreactive IGF-I did not differ between the two compartments (*P* = 0.13). Levels of immunoreactive IGF-II were, on the other hand, decreased in ascites (*P* < 0.001), and the same was true for pro-IGF-II (*P* < 0.001) and IGFBP-3 (*P* < 0.001). By contrast, ascites contained 44% higher levels of IGFBP-2 than serum (*P* < 0.001). Paired individual values of IGFBP-2 in serum and ascites are shown in Figure 1C. Finally, ascites showed a small, but significantly higher degradation of IGFBP-2 than serum (*P* < 0.05), whereas the degradation of IGFBP-3 in serum and ascites was comparable.

### Spearman rank order correlation

For most of the peptides, i.e. total IGF-I, total IGF-II, pro-IGF-II, IGFBP-2 and IGFBP-3, positive correlations were observed when comparing levels in serum/plasma and ascites. By contrast, no correlations were observed for bioactive IGF, IGFBP-4 or PAPP-A (Table 2). No significant correlations between IGF variables and clinical characteristics were observed (data not shown).

**Table 2:** Spearman rank order correlations between concentrations in ascites and serum/plasma

IGF related peptide	Correlation between concentrations in ascites vs. serum/plasma r-value; *P*-value
Bioactive IGF (μg/l)	*r* = 0.31; NS (0.16)
Total IGF-I (μg/l)	*r* = 0.74; <0.001
Total IGF-II (μg/l)	*r* = 0.71; <0.001
Pro-IGF-II (μg/l)	*r* = 0.50; <0.02
IGFBP-2 (μg/l)	*r* = 0.50; <0.02
IGFBP-3 (μg/l)	*r* = 0.85; <0.001
PAPP-A (μg/l)	*r* = −0.04; NS
Intact IGFBP-4 (μg/l)	*r* = 0.09; NS

All measurements were performed in serum/plasma and ascites. Each correlation is based on 22 paired observations. The only exception is the correlation for IGFBP-4, which is based on 18 paired observations. NS: not significant. Only *P*-values below 0.20 are listed.

### *In vitro* experiments in ascites

**Figure 2:**
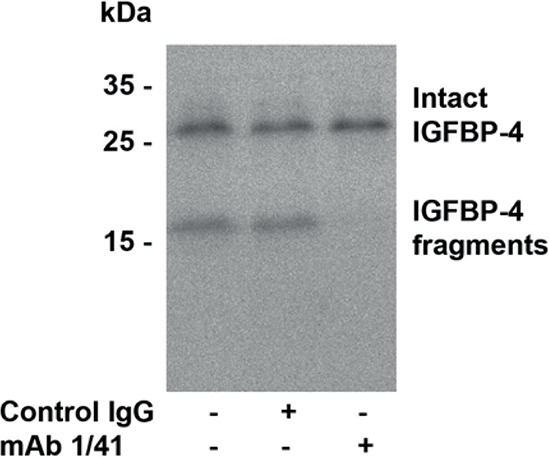
Immunoblotting of IGFBP-4 Ascites was incubated with ^125^I-labelled IGFBP-4 and excess IGF-II in the presence or absence of a PAPP-A inhibiting antibody. As shown, when the PAPP-A inhibiting antibody was added, no degradation of IGFBP-4 was detected.

Our observations suggested the increased IGF bioactivity in ascites to be causally linked to PAPP-A and its cleavage of IGFBP-4. To test this hypothesis, we performed two *in vitro* experiments. The first experiment examined the ability of ascites PAPP-A to cleave radiolabeled IGFBP-4 in the presence of excess IGF-II and in the absence or presence of the PAPP-A inhibitory antibody (mAb 1/41; for further information please refer to [21]). This experiment demonstrated that ascites PAPP-A was able to cleave radiolabeled IGFBP-4. In contrast, when PAPP-A was co-incubated with MAb 1/41, no proteolysis of IGFBP-4 was detectable (Figure 2).

**Figure 3:**
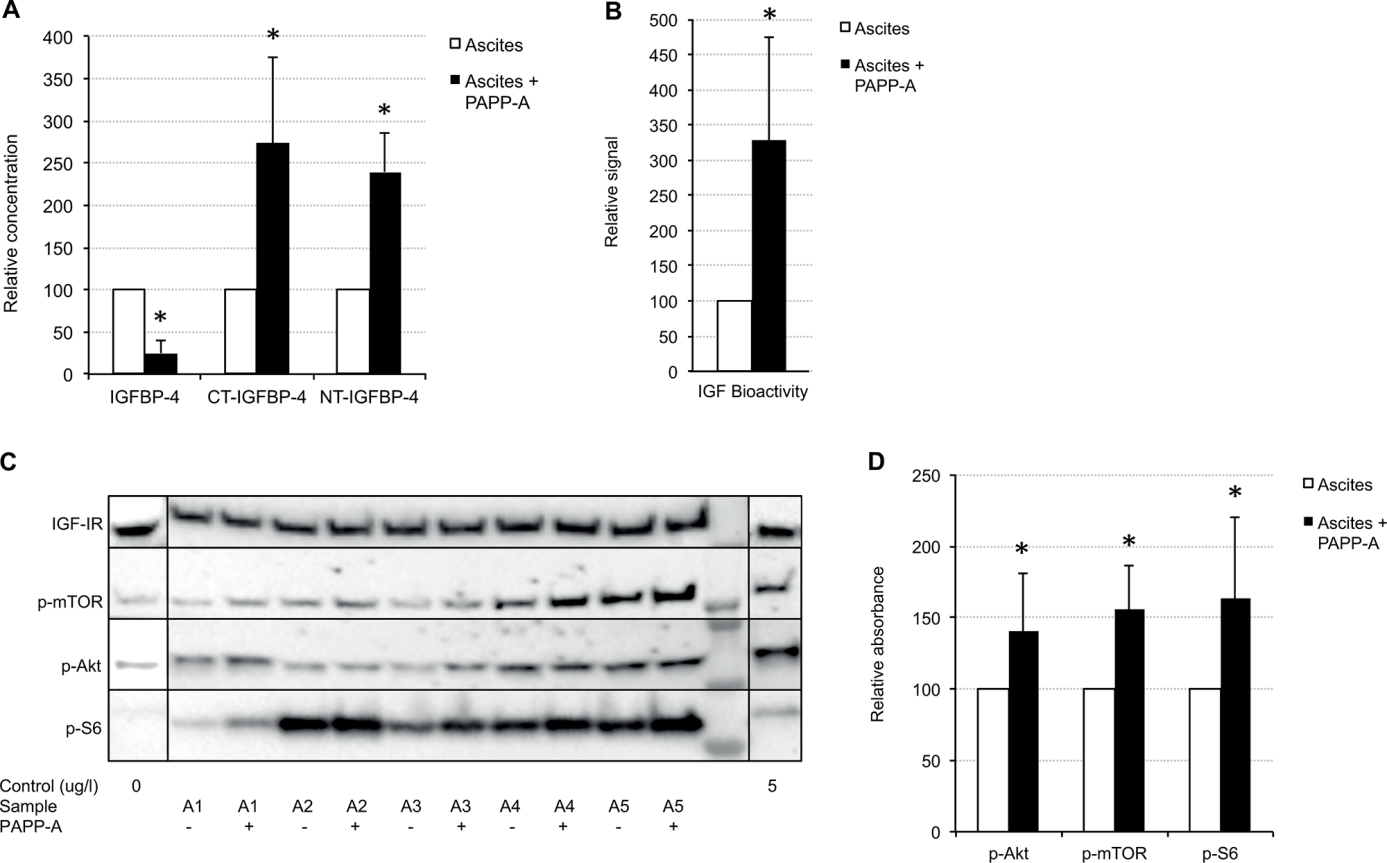
Ascites incubated with or without PAPP-A Ten ascites samples were incubated with either PAPP-A or buffer at 37°C for 6 h. Reaction mixtures were used for stimulation of cells in the KIRA assay. After stimulation for 15 min, cells were lysed and lysates were used for Western blotting. **A.** Relative concentrations of IGFBP-4, C-terminal (CT) IGFBP-4 and N-terminal (NT) IGFBP-4 measured by TR-IFMAs. **B.** Relative IGF bioactivity measured by the KIRA bioassay. **C.** Immunoblotting of cell lysates. Levels of total IGF-IR, p-TOR, p-Akt and p-S6 were determined on the membrane. Buffer incubated with rhIGF-I (0 or 5 ug/l) served as controls. PAPP-A added to rhIGF-I (5 ug/l) had no effect on the phosphorylation beyond that of rhIGF-I (5 ug/l) alone (data not shown). Five ascites samples are shown for illustrative purposes. **D.** Quantification of all western blotting results. Data are presented as mean and standard deviation. *P < 0.05, when comparing ascites with and without PAPP-A.

The second *in vitro* experiment examined whether addition of PAPP-A to ascites was able to increase the degradation of IGFBP-4 and subsequently, to increase the ability of ascites to phosphorylate the IGF-IR. For this purpose, we selected ten ascites samples that contained the highest concentrations of intact IGFBP-4. The experiment demonstrated that addition of recombinant PAPP-A to ascites reduced the amount of intact IGFBP-4 *in vitro* to 25 ± 15% (*P* < 0.01) of the initial concentration and increased the generation of the two IGFBP-4 fragments specifically generated by PAPP-A; the C-terminal and N-terminal IGFBP-4 cleavage fragments by 174 ± 102% (*P* < 0.05) and 134 ± 46% (*P* < 0.01), respectively, as determined by specific immunoassays (Figure 3A). In addition, the degradation of IGFBP-4 increased the ability of ascites to phosphorylate the IGF-IR as compared to ascites incubated with buffer (229 ± 146%, *P* < 0.05; Figure 3B). Finally, we performed immunoblotting of intracellular proteins from the cell line used to measure bioactive IGF. These experiments demonstrated that when PAPP-A was added to ascites, phosphorylation of Akt (40 ± 40%, *P* < 0.05), mTOR (56 ± 30%, *P* < 0.001) and S6 (64 ± 56%, *P* < 0.01) was increased as compared to cells treated with ascites alone (Figure 3C and 3D). Control incubation mixtures containing rhIGF-I or rhIGF-I plus PAPP-A resulted in similar phosphorylations of downstream proteins, whereas buffer alone resulted in limited activation of the signalling proteins (data not shown). Thus, PAPP-A contained no intrinsic IGF-IR activation potential *in vitro*. Levels of total IGF-IR were the same in the cell lysates (data not shown).

### Immunohistochemistry

**Figure 4:**
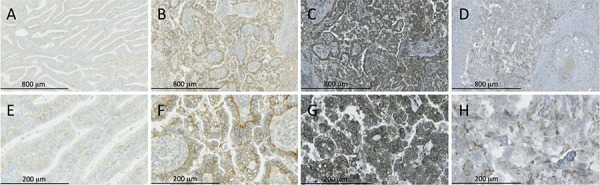
IGF-IR, PAPP-A and IGFBP-2 immunohistochemistry of ovarian tumorsRepresentative images are shown The ovarian tumor sections showed a variable expression of IGF-IR (low expression, panel A+E, high expression, panel B+F). The staining for PAPP-A showed a high expression in all tumor specimens (panel C+G), whereas the staining for IGFBP-2 showed moderate positivity (panel D+H). The bars indicate 800 microns **A–D**, and 200 microns **E–H**, respectively. For technical reasons the bar size differs from that in Figure 5.

**Figure 5:**
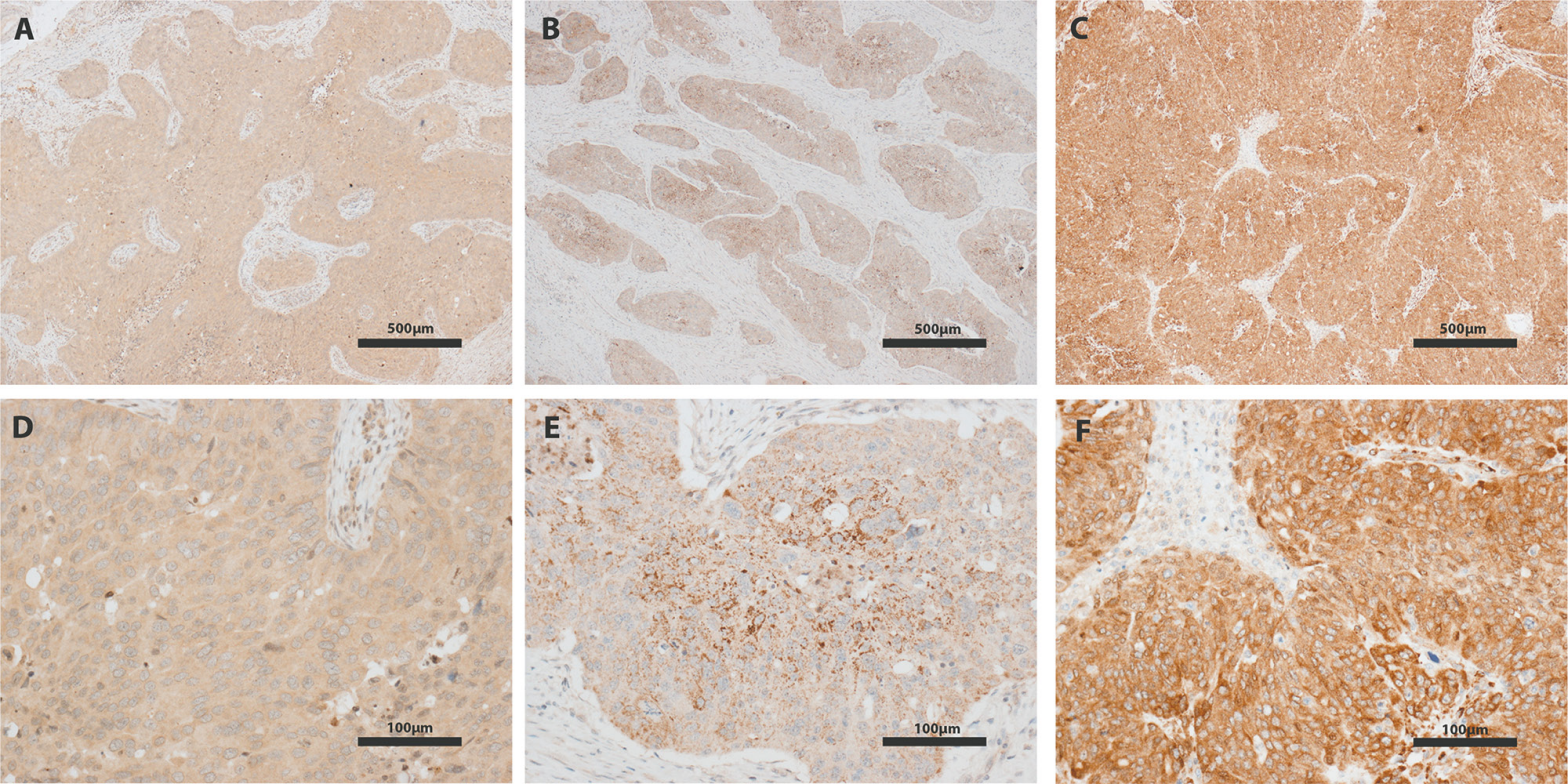
IGF-I, IGF-II and IGFBP-4 immunohistochemistry of ovarian tumors Representative images are shown. IGF-I (panel A+D) displayed a weak cytoplasmic staining in all 17 tumors. However, all sections also showed variable faint expression of IGF-I in tissue surrounding the tumor. This could be specific staining of stroma and/or inflammatory cells. All 17 tumors showed a weak, granular cytoplasmic expression of IGF-II (panel B+E) with foci of coarse granules at perinuclear or apical, sub-membranous locations. IGFBP-4 (panel C+F) showed a granular cytoplasmic staining pattern with focal perinuclear accentuation. In a few cases, a nuclear staining pattern was seen focally. The expression of IGFBP-4 varied from weak to strong, with the majority of cases showing a strong, extensive staining. The bars indicate 500 microns **A–C,** and 100 microns **D–F,** respectively. For technical reasons the bar size differs from that in Figure 4.

Immunohistochemistry on tumors (*n* = 19) removed during surgery documented the presence of IGF-IR in all specimens but one. Two examples of IGF-IR immunostaining are shown in Figure 4 (panel A, B, E and F), illustrating the variability of IGF-IR staining across the 18 positive tumors (low and extensive staining, respectively). Tumors showed IGF-IR staining related to the cell membranes. Staining for PAPP-A (Figure 4, panel C and G) showed an apparent high level of protein expression in all tumors with a similar cell membrane accentuated staining pattern, whereas the staining for IGFBP-2 (Figure 4, panel D and H) showed a granular cytoplasmic staining pattern in all specimens. Staining for IGF-I, IGF-II and IGFBP-4 was evaluated in 17 out of 19 tumors. IGF-I (Figure 5, panel A and D) displayed a weak cytoplasmic staining in all 17 tumors. However, all sections also showed variable faint expression of IGF-I in the tissue surrounding the tumor. This could be specific staining of stroma and/or inflammatory cells. All 17 tumors showed a weak, granular cytoplasmic expression of IGF-II (Figure 5, panel B and E) with foci of coarse granules at perinuclear or apical, sub-membranous locations. The presence of IGFBP-4 (Figure 5, panel C and F) was observed in all 17 tumors, showing a granular cytoplasmic staining pattern with focal perinuclear accentuation. In a few cases, a nuclear staining pattern was seen focally. The expression of IGFBP-4 varied from weak to strong, with the majority of cases showing a strong, extensive staining.

## DISCUSSION

We have previously demonstrated that non-malignant ascites contains increased *in vitro* IGF bioactivity as compared to serum [8]. We therefore hypothesized that also malignant ascites has higher IGF bioactivity than serum. The present study confirmed this hypothesis as the IGF bioactivity of ascites from women with ovarian cancer was 31% higher than that of serum. Our study also provided a mechanistic explanation for this finding involving PAPP-A and its cleavage of IGFBP-4, which serves to release bioactive IGF. As PAPP-A and IGFBP-4 were contained in ascites and also within the tissue, this suggest that both soluble PAPP-A and tissue-associated PAPP-A may function to increase IGF action. Finally, by demonstrating the presence of the IGF-IR on the tumors, we provided a pathogenic link between our findings in ascites and the ovarian tumors.

Ascites contained a 46-fold higher concentration of PAPP-A than serum. Further, PAPP-A was detectable on all tumors by immunohistochemistry. This indicates that the tumors are producing and secreting PAPP-A into the abdominal cavity. Whether the same is true for IGFBP-4, the primary target of PAPP-A, is less certain. However, all tumors contained IGFBP-4. Thus, we speculate that the tumors, by secretion of PAPP-A and IGFBP-4, are able to regulate IGF action locally.

Following measurement of PAPP-A/proMBP complexes in ascites, we estimated that approximately 80% of the PAPP-A was enzymatically active. The only known function of PAPP-A is to act as an IGFBP cleaving enzyme. The primary target of PAPP-A is IGFBP-4, which is cleaved in an IGF-dependent manner, i.e. IGFBP-4 is only cleaved when it carries either IGF-I or IGF-II. Consequently, IGFBP-4 serves as an IGF donor in the presence of PAPP-A [13, 14]. In the present study, we had access to assays specifically targeting intact IGFBP-4 and the two IGFBP-4 fragments generated after PAPP-A-mediated cleavage [22, 23]. By these assays we demonstrated that ascites contained higher levels of C-terminal IGFBP-4 fragments than intact IGFBP-4. Furthermore, our *in vitro* experiments demonstrated that ascites cleaves IGFBP-4, but only in the absence of a PAPP-A inhibiting antibody [21]. Additionally, when PAPP-A was added to ascites, this increased IGFBP-4 degradation, IGF-IR activation and phosphorylation of intracellular proteins involved in the IGF-IR signaling pathways. However, PAPP-A added to samples containing rhIGF-I, but no IGFBP-4, did not increase IGF-IR activation or the intracellular signaling cascade. In conjunction, our findings yield evidence that the increased IGF bioactivity in ascites is at least partially explained by a PAPP-A-mediated cleavage of IGFBP-4 resulting in release of IGF.

For an elevated *in vitro* IGF bioactivity to be of pathogenic relevance, target cells are required to express the IGF-IR. Therefore, tumors were examined by immunohistochemistry, which confirmed the presence of the IGF-IR on all tumors but one. This observation is in agreement with findings by others [24, 25]. It supports the notion that ascites may serve as a tumor-stimulating medium, acting via the IGF-IR. Furthermore, as previously demonstrated all tumors expressed IGF-I and IGF-II peptide [3, 24, 25]. However, we cannot say whether the tumor IGFs originate from an uptake by the IGF-IR and/or a local production within the tumors.

In mice, transplantation of SKOV3 cells overexpressing PAPP-A resulted in an accelerated tumor growth [12], most likely by promoting local IGF bioavailability through cleavage of IGF:IGFBP-4 complexes at the cell surface [13]. This mechanism may also be operative in humans. Our study suggests that soluble PAPP-A may increase levels of bioactive IGF in ascites, whereas tumor-assocated PAPP-A may increase IGF bioactivity locally at the cell membrane [26]. The relative contribution of these two mechanisms is difficult to estimate in a clinical setting.

Our findings suggest that PAPP-A controls the ability of ascites to activate the IGF-IR and, accordingly, tumor growth. This idea gains support from a recently published experimental investigation by Becker *et al*. [16]. The authors studied the effect of an intraperitoneal administered PAPP-A neutralization antibody on ascites formation and tumor growth in mice receiving ovarian carcinoma tumor grafts from patients. It was demonstrated that an antibody-mediated neutralization of PAPP-A reduced intraperitoneal tumor growth as well as delayed or inhibited formation of ascites [16]. Thus, accumulating evidence supports a direct pathophysiological link between tumor growth, ascites and its content of PAPP-A in ovarian carcinoma.

PAPP-A does not cleave IGFBP-3. Accordingly, the degradation of IGFBP-3 demonstrated in ascites and serum from the patients requires the presence of another proteinase specific for this binding protein [27]. As for IGFBP-4, proteolytic degradation of IGFBP-3 increases the level of bioactive IGF [28]. Therefore, we cannot conclude that PAPP-A is the only protease responsible for the increase in bioactive IGF. However, so far PAPP-A is the only protease that has been directly linked to ovarian cancer.

Many ovarian tumors express IGFBP-2 [29, 30]. Furthermore, serum IGFBP-2 levels are characteristically elevated in ovarian cancer patients and correlate with the stage of disease as well as with the prognosis [30–33]. In combination, these findings suggest that IGFBP-2 is involved in the pathogenesis of ovarian carcinoma and, consequently, it may serve as a circulating biomarker [33]. These findings were extended by the present study. Immunohistochemistry showed that all tumors examined expressed IGFBP-2. Immunoassay showed that IGFBP-2 levels were close to 50% higher in ascites than serum. Given that levels of IGFBP-2 in ascites and serum were positively correlated, our findings support the concept that the elevated serum levels are attributable to an increased production of IGFBP-2 by the tumor [30]. If this interpretation is correct, it implies that IGFBP-2 is able to diffuse from the ascites compartment to the circulation. Interestingly, IGFBP-2 is a substrate of PAPP-A, being cleaved in an IGF-dependent manner [34]. However, despite elevated ascites levels of PAPP-A, the degradation of IGFBP-2 was modest. Two explanations are likely. Firstly, compared to IGFBP-4, IGFBP-2 is a poor substrate for PAPP-A [35]. Secondly, the PAPP-A-mediated cleavage of IGFBP-2 requires that the binding protein is associated with its ligand. Thus, we speculate that the major fraction of IGFBP-2 is not saturated with IGF. This, however, needs further confirmation.

Experimental studies have demonstrated an effect of IGF-I and the IGF-IR in all stages of cancer development. This includes transformation of malignant tissue, tumor growth and metastasis as well as sensitivity to chemotherapy [1–4, 36]. Nevertheless, the majority of clinical trials with IGF-I and IGF-IR inhibiting drugs have been disappointing [1, 2, 37]. However, cancer is a highly heterogeneous disease and even within the same type of malignancy, the affected molecular pathways may differ [38]. On this basis, some have argued for the need for predictive biomarkers that can identify patients more likely to respond to anti IGF-I treatment and/or to exclude patients more likely to experience side effects [37]. In this context some of our patients demonstrated considerably higher ascites than serum levels of PAPP-A, IGFBP-2 and bioactive IGF. We therefore suggest that the biomarker potential of these molecules are analyzed in larger cohorts. This may lead to improved selection of patients eligible for treatments targeting IGF-I and/or the IGF-IR.

The present study has limitations. Firstly, we have no nutritional information (e.g. BMI, serum albumin or body composition) of our patient group. Instead, we compared patients with a group of age-matched healthy women by the use of the circulating IGF system, which is a sensitive marker of nutrition and systemic illness [17]. This comparison revealed differences in regards to total IGF-I and IGFBP-3, whereas bioactive IGF remained similar in the two groups. The latter is more sensitive to fasting than total IGF-I and IGFBP-3 [39, 40], and accordingly, patients are unlikely to be catabolic. Nevertheless, we cannot exclude that some of the differences in the IGF system components that were observed in ascites as well as in serum/plasma in the patient group may be secondary to nutritional changes rather than to the presence of an ovarian carcinoma. Secondly, we only included 22 patients and this sample size precluded a comparison of different cancer subtypes. Thirdly, we have measured IGF bioactivity by an *in vitro* method, which does not necessarily reflect the true, endogenous activity of the IGF system in humans. Finally, the bioassay does not allow discrimination between IGF-I- and IGF-II-induced IGF-IR activation. We are aware of this and accordingly, the read-out of the assay has been designated “bioactive IGF”. In ascites, the concentration of total IGF-II was approximately 4-fold higher than that of total IGF-I, hereby indicating that IGF-II may contribute significantly to IGF-IR activation. On the other hand, IGF-II only has an IGF-IR cross-reactivity of 12% relative to IGF-I. Hence, it is reasonable to assume that the majority of the signal from the bioassay originates from IGF-I [41].

In conclusion, we demonstrate that ascites from patients with ovarian cancer contain a significantly greater potential to activate the IGF-IR *in vitro* than the corresponding serum samples. Given that the concentration of IGF-I and IGF-II in ascites was unchanged and reduced, respectively, as compared to serum, this points to an overall reduction of the IGF-binding capacity in ascites. This may be secondary to increased IGFBP proteolytic activity via PAPP-A and possibly by other proteases. Notably, the ability of PAPP-A to increase bioactive IGF in ascites was supported by *in vitro* experiments. In conjunction with immunohistochemistry of the tumors, which identified the presence of the IGF-IR, PAPP-A and IGFBP-4, our data suggest that PAPP-A may increase IGF bioactivity in solution as well as at the surface of the tumors. On the basis of our findings, we hypothesize that the assessment of PAPP-A present in ascites may be a novel approach to identify patients in whom drugs targeted against the IGF system may be of clinical value.

A present in ascites may be a novel approach to identify patients in whom drugs targeted against the IGF system may be of clinical value.

## MATERIALS AND METHODS

### Study participants

The patient group consisted of women suffering from ovarian or peritoneal cancer with accumulation of ascites. From October 2011 to June 2012 patients were enrolled from the Department of Gynecology, Aarhus University Hospital, Denmark. From February 2012 to June 2012 we also enrolled patients from Clinic of Gynecology, Juliane Marie Centre, Rigshospitalet, Copenhagen. By the end of June 2012, the study population consisted of 30 patients from Aarhus University Hospital and four patients from Rigshospitalet. Thirty patients were newly diagnosed whereas four patients had received neo-adjuvant treatment before surgical debulking. Among the latter four, ascites was collected after three series of chemotherapy (*n* = 1) or prior to commencement of chemotherapy (*n* = 3). Otherwise, ascites was collected peri-operatively. In all cases, serum and EDTA plasma were sampled prior to surgery and chemotherapy. Of 34 patients, 22 (age 62 [55–73] years, median [interquartile range]) fulfilled inclusion criteria: ascites due to ovarian or peritoneal cancer and the availability of both ascites and serum.

Fifteen age-matched healthy women (age 63 [62–76] years) were included. They received no medication and blood samples were drawn after an overnight fast. All participants provided written informed consent after receiving written and oral information regarding the study according to the Helsinki Declaration. The study was approved by the Local Ethics Committees in Aarhus as well as in Copenhagen. Data on bioactive IGF in controls have recently been published elsewhere [42].

### Measurement of *in vitro* IGF bioactivity

The *in vitro* IGF-IR activation was assessed by an in-house kinase receptor activation (KIRA) assay, performed as originally outlined [41], with recent modifications [39]. The KIRA assay is designed to quantify the ability of a given sample to phosphorylate the IGF-IR in cultured HEK 293 cells transfected with cDNA encoding the human *IGF-IR gene.* Thus, the assay takes into consideration the ability of IGFBPs and IGFBP-proteases to modulate the concentration of IGF accessible to the receptor; for details please refer to our previous publications [41, 43]. Signals from the samples were compared to that of a serial dilution of rhIGF-I (WHO international standard 02/254). Accordingly, the KIRA assay signal was expressed in μg/l. In addition to IGF-I, the KIRA assay also detects IGF-II and pro-IGF-II activation of IGF-IR, which have a reactivity of 12% and 2%, respectively, of that of IGF-I, whereas proinsulin, insulin and insulin analogues barely interact (<1%). To acknowledge the fact that both IGF-I and IGF-II can activate the IGF-IR *in vivo* as well as *in vitro*, the output of the assay has been designated “bioactive IGF”. The KIRA assay has a detection limit of approximately 0.1 μg/l and intra-assay coefficient of variation (CV) of 4% and 8% for signals and corresponding concentrations, respectively. The inter-assay CV of a control serum sample is 15%.

### Separation of IGF-I, IGF-II and pro-IGF-II

The IGFBP profile of ascites differs substantially from that of serum. Therefore, IGF-I, IGF-II and pro-IGF-II were separated from the IGFBPs by size exclusion fast protein liquid chromatography (FPLC) at low pH. This methodology is considered to be the gold standard for removal of IGFBPs [44]. Although laborious in nature, this method is the best way to ensure full separation of IGFs and IGFBPs in samples with a highly abnormal composition of IGFBPs such as ascites. In brief, serum (100 μl) or ascites (300 μl) was incubated with 1 M acetic acid to a total volume of 1000 μl which ensured a pH < 2.3. After at least 30 min of incubation at low pH, samples were fractionated at a flow rate of 1 ml/min on a Superdex 75 10/300 column (GE Healthcare, Uppsala, Sweden) equilibrated with running buffer (0.2 M acetic containing 0.05% Tween 20), using a pump (Smartline 1000, Knauer, Berlin, Germany). Sample delivery to the column was performed by an autosampler (model 3800, Knauer, Berlin, Germany). Fraction collection was performed using a Fraction Collector CHF122SB (Advantec, Dublin, CA, USA). For further details please refer to [45].

### Immunoassays for total IGF-I, total IGF-II and pro-IGF-II

The fractions containing mature IGF and pro-IGF-II were assayed by in-house time-resolved immunofluorometric assays (TR-IFMAs) developed and validated in our laboratory, as recently detailed [45]. In brief, the IGF-I assay was calibrated against the international IGF-I standard (WHO 02/254). Neither IGF-II nor pro-IGF-II showed any cross-reactivity in the IGF-I assay. All samples were assayed in duplicate with an intra-assay CV averaging 2%. The inter-assay CV (including FPLC and immunoassay) of an IGF-I calibrator and a control serum sample averaged 8 and 13%, respectively. The IGF-II assay was calibrated against the international IGF-II standard (WHO 96/538). IGF-I did not cross react whereas pro-IGF-II cross-reacted by 50% in the IGF-II assay. The intra-assay CV of samples assayed in duplicate averaged 2%. The inter-assay CV of an IGF-II calibrator and a control serum sample averaged 13% and 11%, respectively. Pro-IGF-II was measured by specific assay using recombinant pro-IGF-II (GroPep, Adelaide, Australia) as calibrator. Neither IGF-I nor IGF-II showed any cross-reactivity in the pro-IGF-II assay. All samples were assayed in duplicate with an intra-assay CV averaging 2%. The inter-assay CV of a pro-IGF-II calibrator and a control serum sample both averaged 11%.

### Immunoassays for IGFBP-2, IGFBP-3 and PAPP-A

IGFBP-2 was determined by an in-house TR-IFMA as previously described [46]. Intra- and inter-assay CVs averaged 5% and 12%. IGFBP-3 was determined by a commercial kit (IS-4400) from Immunodiagnostic Systems (IDS), using an automated immunoassay system (iSYS) as recently published [47]. PAPP-A was determined by a commercial ELISA kit (Ansh Labs, catalogue no. AL-101), generously provided by Ansh Labs (Webster, TX, USA).

### Immunoassays for intact IGFBP-4 and IGFBP-4 fragments

Ascites and EDTA-plasma levels of IGFBP-4, and the C- and N-terminal IGFBP-4 fragments were determined by novel in-house sandwich TR-IFMAs based on monoclonal antibodies (IgG) and recombinant calibrators generously provided by HyTest Ltd. (Turku, Finland). Detection limits were 0.5 μg/l, 0.4 μg/l and 0.9 μg/l for intact IGFBP-4, C-terminal IGFBP-4 and N-terminal IGFBP-4 immunoassays, respectively. In all three assays, inter- and intra-assay CVs were less than 15% and 10%, respectively. The assays were performed as recently described [22]. Due to limited amounts of paired ascites and plasma, only intact IGFBP-4 and C-terminal IGFBP-4 were measured in all patient samples.

### Determination of enzymatically active PAPP-A in ascites

PAPP-A exists in two major forms: an enzymatically active form corresponding to free, dimeric PAPP-A, and an enzymatically inactive form, composed of dimeric PAPP-A covalently linked to two molecules of eosinophil major basic protein (proMBP) [18, 19]. Therefore, to estimate the proteolytical activity of PAPP-A in ascites, we determined the concentration of PAPP-A/proMBP complex and expressed that as a fraction of total PAPP-A levels. The PAPP-A/proMBP complex was determined as previously described [20].

### Cleavage of radiolabeled IGFBP-4 by PAPP-A in ascites

Purified IGFBP-4 [48] was labeled with ^125^I (Amersham Biosciences, Hillerød, Denmark), and cleavage reactions were carried out by incubation of ascites samples (6 μl) with 10 nM ^125^I-IGFBP-4 and 100 nM IGF-II (GroPep Bioreagents, Adelaide, Australia) in 50 mM Tris-HCl, 100 mM NaCl, 1 mM CaCl_2_, pH 7.5 [35]. The total reaction volumes were 30 μl. Following 4 h of incubation at 37°C, the reactions were terminated by the addition of hot SDS-PAGE sample buffer supplemented with 25 mM EDTA. Substrate and cleavage products were separated by 12% SDS-PAGE and visualized by autoradiography using a storage phosphor screen (Molecular Dynamics, Sunnyvale, CA) and a Typhoon imaging system (GE Healthcare, Brøndby, Denmark). Prior to incubation, ascites samples were mixed and pre-incubated with PAPP-A inhibitory antibody (mAb 1/41, 50 μg/ml) [21] or an irrelevant isotype control antibody and incubated for 20 min.

### Effect of exogenous PAPP-A on ascites IGFBP-4 fragmentation and IGF-IR signalling

The PAPP-A-mediated cleavage of endogenous IGFBP-4 in ascites was further assessed by incubating ascites samples (*n* = 10) with 0.1 nM recombinant PAPP-A [19] or buffer at 37°C for 6 h. Ascites samples containing the highest amounts of intact IGFBP-4 were chosen. Buffer containing rhIGF-I (0 or 5 ug/l) or rhIGF-I (5 ug/l) and PAPP-A (0.1 nM) were incubated and served as controls. The reaction mixtures were immediately used for measurements of bioactive IGF using the KIRA bioassay. In addition, IGFBP-4, C-terminal and N-terminal IGFBP-4 were determined in the reaction mixtures using immunoassays as previously described [22, 23]. Finally, to assess the intracellular signalling pathways initiated by IGF-IR activation, cell lysates from the IGF bioactivity measurements were separated by 4–15% SDS-PAGE and transferred to PVDF membranes. Levels of phosphorylation of the intracellular proteins Akt, mTOR and S6 were determined by probing the blots with anti-p-Akt antibody (AF887, R&D Systems, Abingdon, UK), anti-p-TOR antibody (AF1665, R&D Systems) and anti-p-S6 antibody (AF3918, R&D Systems). Total IGF-IR levels in the cell lysates were determined using anti-hIGF-IR antibody (MAB391, R&D Systems) and used as loading controls. Additionally, stain-free total protein quantitation using the ChemiDoc™ system (Bio-Rad) served as total protein loading control.

### IGFBP immunoblotting

Standard Western blotting techniques were applied and results were analyzed on a Bio-Rad platform (Copenhagen, Denmark). In brief, serum and ascites were diluted 1:40 in laemmli buffer containing 5% beta-mecaptoethanol (Bio-Rad), heated to 94°C for 15 min and thereafter left to cool at RT. Samples were loaded in duplicate (25 μl per lane) and protein separated on midi format stain-free SDS gels (12% Bis-Tris SDS gel, Criterion™ TGX, Bio-Rad), transferred to a PVDF membrane (Trans-Blot ^®^Turbo, Bio-Rad) and immunoblotted. Total protein on gels and blots were visualized by activation of the gel for 5 min with UV-light using the ChemiDoc™ system (Bio-Rad). Blots were probed with polyclonal antibodies against human IGFBP-2 (unlabeled, 0.05 mg/l; sc-6001, Santa Cruz Biotechnology, Dallas, TX) or IGFBP-3 (biotinylated, 0.05 mg/l; BAF675, R&D Systems), followed by incubation with HRP-anti-goat antibody for IGFBP-2 detection (HAF017, R&D Systems) or HRP-streptavidin for IGFBP-3 detection (4800–30-06, R&D Systems) and developed using chemiluminescence (SuperSignal^®^West Dura, Thermo Scientific, Hvidovre, Denmark). Images were analyzed using Image Lab 4.0.1 (Bio-Rad) and mean intensities were calculated and used for semi-quantitative analysis. Intact IGFBP-2 appeared as a 32 kilo Dalton (kDa) band, and its fragments as 22 and 18 kDa bands. Intact IGFBP-3 appeared as a double band at 38 and 42 kDa, whereas the main fragmented bands corresponded to 22–23 kDa, 17–18 kDa, and 15–17 kDa, respectively. To yield an estimate of IGFBP-2 or -3 degradation, the sum of intensities of the fragmented bands was expressed as a percentage of the total intensity of the IGFBP of interest.

### Immunohistochemistry

Immunohistochemistry was performed by standard techniques. In brief, formalin-fixed paraffin-embedded ovarian tumor specimens from 19 patients were sectioned at 2 microns and mounted on glass slides. Primary antibodies were directed against IGF-1R (clone G11, Ventana Medical Systems, Tucson, AZ, USA) diluted 1/100; PAPP-A (PAC1-D8-mIgG2a) [49] diluted 1/200; IGFBP-2 (Rabbit Polyclonal #3922, Cell Signaling Technology, Boston, MA, USA) diluted 1/25; IGF-I (ab40657, Abcam Cambridge, UK) diluted 1/200; IGF-II (ab9574, Abcam diluted 1/200 and IGFBP-4 (ab83846, Abcam) diluted 1/500. Immunohistochemistry was performed using a Benchmark XT automated stainer (Ventana Medical Systems). Deparaffinisation, epitope retrieval, and immunostaining were performed according to the instructions of the manufacturer. Binding of antibody was visualized with the ultraVIEW Universal diaminobenzidine detection system (Ventana Medical Systems). The sections were counterstained with Mayers haematoxylin, dehydrated and mounted using hydrophobic mounting medium (Leica Microsystems, Wetzlar Germany). Whole tissue sections were scanned at a maximum resolution of 40 x using a whole slide scanner (NanoZoomer 2.0, Hamamatsu, Japan). Digital images were then imported into Adobe Illustrator.

### Statistics

Comparisons of patients and healthy women were performed using Mann-Whitney's rank sum test. Paired comparisons of ascites and serum were analyzed by paired *T*-test or Wilcoxon's signed rank test. Data are presented as mean and standard deviation or median and interquartile range. Possible associations between IGF variables and disease characteristics were examined by a linear regression model with FIGO stage or tumor grade as continuous and explanatory variables. Associations between IGF system components were evaluated by Spearman rank order correlation analysis. *P*-values < 0.05 were considered to be statistically significant.
